# Clinical Manifestations and Genetic Profile of Chinese Patients with NK-Cell Large Granular Lymphocytic Leukemia—A Single-Center Retrospective Analysis

**DOI:** 10.3390/ijms27146227

**Published:** 2026-07-13

**Authors:** Zhe Zhuang, Huiying Zhu, Chao Chen, Yiao Di, Zhangyuting He, Wei Zhang, Daobin Zhou, Yan Zhang

**Affiliations:** 1 Department of Hematology, Peking Union Medical College Hospital, No. 1 Shuaifu Yuan, Beijing 100032, China; zhuangzhe@pumch.cn (Z.Z.);; 2 Department of Internal Medicine, Peking Union Medical College Hospital, No. 1 Shuaifu Yuan, Beijing 100032, China

**Keywords:** natural killer cells, large granular lymphocyte, leukemia, *STAT3*

## Abstract

Natural killer cell large granular lymphocytic leukemia (NK-LGLL) is a rare and heterogenous lymphoproliferative disorder. This study retrospectively evaluated 35 consecutive Chinese patients (median age 58 years) to evaluate their unique clinical–biological profiles and treatment responses. Our Chinese population exhibited a distinct comorbidity spectrum, characterized by a lower prevalence of concurrent arthritis (2.9%) and secondary malignancies, compared with Western cohorts. At diagnosis, 31.4% of the cohort had neutropenia, 42.9% had anemia, and 31.4% had thrombocytopenia. The median large granular lymphocyte count was 3.9 × 10^9^/L (range 0.11–114.8 × 10^9^/L; IQR 1.9 × 10^9^/L, 5.9 × 10^9^/L). Immunophenotyping consistently identified as a CD3- CD56+ clone. Notably, genomic profiling via NGS revealed a *STAT3* mutation rate of 14.3%. Regarding therapeutic efficacy, frontline immunosuppressive therapy with cyclophosphamide or cyclosporine was associated with favorable clinical responses (best overall response, complete remission rate 66.7% for both). Additionally, sirolimus emerged as a potentially highly effective salvage option, yielding an overall response rate of 85.7% (95%CI 42.1–99.6%) and complete remission rate of 57.1%. With an estimated 3-year overall survival rate of 85.6% (95%CI 73.3%, 99.8%), our findings suggest a generally indolent clinical course of NK-LGLL in this Chinese cohort and highlight the potential of mTOR inhibition in refractory cases, warranting further prospective investigation.

## 1. Introduction

Large granular lymphocytic leukemia (LGLL) is a rare lymphoproliferative disorder characterized by the persistent (>6 months) abnormal clonal expansion of lymphocytes exhibiting distinct morphological features, known as large granular lymphocytes (LGLs), in the peripheral blood, bone marrow, and spleen [1,2]. According to the World Health Organization (WHO)-HAEM5 classification, LGLL is categorized into three entities based on the lineage of the leukemic cells and genetic profile: T-cell large granular lymphocytic leukemia (T-LGLL); natural killer cell large granular lymphocytic leukemia (NK-LGLL), which was formerly designated as chronic lymphoproliferative disorder of NK cells (CLPD-NK) in the WHO-HAEM4R; and aggressive NK-cell leukemia (ANKL) [3]. Approximately 85% of all LGLL cases arise from CD3^+^CD57^+^CD56^−^ T cells, namely T-LGLL, while the remaining 15% originate from CD3^−^CD56^+^ NK cells, namely NK-LGLL [4,5].

Typically, both T-LGLL and NK-LGLL manifest as indolent disease courses, with asymptomatic disease onset and relatively slow progression. The reported median age at diagnosis is 60–65 years. Many patients exhibit asymptomatic lymphocytosis, while common clinical manifestations include chronic cytopenia-related symptoms such as fever and recurrent infections, anemia, thrombocytopenia, fatigue, and B symptoms, as well as a notable prevalence of concurrent autoimmune diseases [2,6,7].

The incidence of LGLL has not been accurately determined, approximately accounting for 2% to 5% of chronic lymphoproliferative disorders in North America and up to 5% to 6% Asia, with an estimated incidence of 0.2 to 0.72 per 1,000,000 person-years in Western populations [8,9], while large-scale prospective or retrospective studies based on LGLL are limited. Only a few have reported features of LGLL in cohorts with sizes greater than 100 [5,7,10,11,12,13,14]; most were Western cohorts and focused on patients with T-LGLL. In contrast to the relatively more characterized T-LGLL, the clinical, biological, and genomic landscapes of NK-LGLL remain understudied, with limited published data available worldwide. The largest cohort for NK-LGLL included 70 patients from Western countries [15]. Large-scale clinical descriptions and outcome analyses of NK-LGLL focusing on Asian populations are notably scarce. This study aimed to delineate the primary clinical–biological characteristics, therapeutic responses, and clinical outcomes of NK-cell large granular lymphocytic leukemia patients diagnosed and treated in China, with the goal of enhancing the understanding of this rare disease entity.

## 2. Results

### 2.1. Clinical Features of NK-Cell Large Granular Lymphocytic Leukemia

Our longitudinal NK-LGLL cohort consisted of 35 patients: 16 females and 19 males. As shown in Table 1, the median age at diagnosis was 58 years (range 30–78 years), with 27 patients (77.1%) older than 50. Disease onset was predominantly insidious. Seventeen patients (48.6%) were asymptomatic at diagnosis, with incidental abnormal blood counts found during routine examinations. Notably, the median diagnostic delay was 12.2 months (range 1–91.3 months), and two asymptomatic patients developed LGLL-related symptoms (fever, arthralgia) 8 months after their diagnoses. Among symptomatic patients (*n* = 18), the median symptom-to-diagnosis interval was 5 months (range 1–146 months). Common clinical manifestations included fatigue (20.0%), recurrent infections (20.0%), bleeding (5.7%), and splenomegaly (8.6%). Notably, 10 patients had active autoimmune diseases before the onset of NK-LGLL, including two cases of autoimmune hemolytic anemia, two cases of systemic lupus erythematosus, one case of primary biliary cholangitis, one case of leukocytoclastic vasculitis, one case of rheumatoid arthritis, and three cases of immune-mediated peripheral neuropathy.

At initial presentation, the median LGL count was 3.9 × 10^9^/L (range 0.11–114.8 × 10^9^/L; IQR 1.9 × 10^9^/L, 5.9 × 10^9^/L). Cytopenia was prevalent, including neutropenia (31.4%, *n* = 11), anemia (42.9%, *n* = 15), and thrombocytopenia (31.4%, *n* = 11).

As for co-existing malignancies (CMs), one patient had a history of prostate cancer, while another was diagnosed with concurrent myelodysplastic syndrome with excess blasts (MDS-EB), which progressed to acute myelogenous leukemia during follow-up.

### 2.2. Characterization of Immunophenotypes

**Figure 1:**
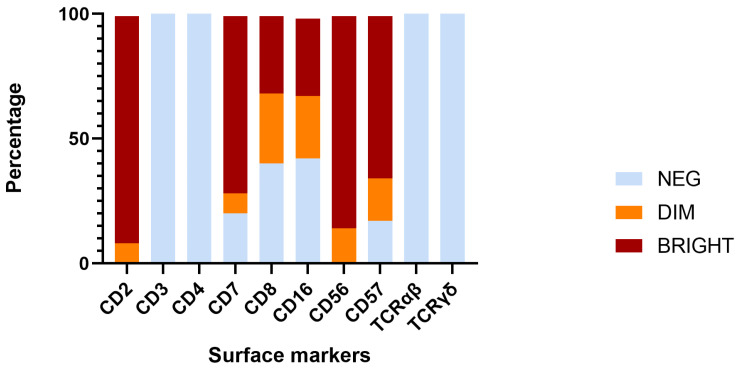
Immunophenotypes of NK-LGLL patients. The immunophenotypes of NK-LGLL patients revealed by flow cytometry are presented. CD2 was bright in 91.43% and dim in 8.57% of the cohort. CD56 was bright in 85.71% and dim in 14.29%. CD57 was bright in 65.72%, dim in 17.14%, and negative in 17.14%. CD7 was bright in 71.43%, dim in 8.57%, and negative in 20.0%. CD8 was bright in 31.43%, dim in 28.57%, and negative in 40.0%. Surface CD16 was bright in 31.43%, dim in 25.71%, and negative in 42.86%. All were negative for CD3 and CD4.

Flow-cytometric analysis of peripheral blood was performed for diagnostic confirmation. Analysis of the flow-cytometric results (Figure 1) suggested that the abnormal NK-cell clone had predominant immunophenotypes of TCRαβ− (100%), TCRγδ− (100%), CD2+/dim (100%), sCD3− (100%), CD4− (100%), CD56+/dim (100%), CD57+/dim (82.9%), and CD7+/dim (80.0%). CD8 expression was dim to bright in 60.0% of NK-LGLL patients, while surface CD16 was positive in 57.1%. Identical immunophenotypes were consistently observed in paired bone marrow samples (*n* = 4).

### 2.3. Characterization of Mutational Status

**Figure 2:**
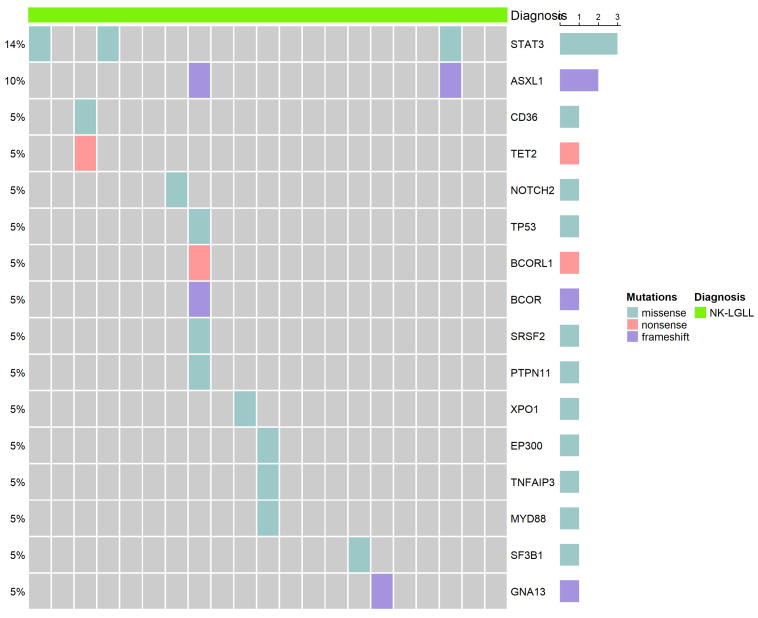
Mutational statuses of NK-LGLL patients. Overview of gene mutations in the NK-LGLL cohort. Mutation types are color-coded as shown in the legend. Gray cells indicate no detected mutations.

Among our NK-LGLL cohort, 21 patients underwent next-generation sequencing. Mutations were detected in 10 cases (47.6%), as shown in Figure 2 (details of mutations presented in Supplementary Table S3). Among those in whom mutations were detected, the majority were oligo-mutated, involving pathways such as JAK-STAT, NF-κB, epigenetic regulation, and RNA splicing. *STAT3* mutations were detected in three (14.2%), including D661Y, N647I, and S614R. *STAT5b* mutation was not detected. Patients with and without detectable mutations had highly comparable clinical features and laboratory findings. Further statistical analysis confirmed that the mutation-positive and -negative cohorts remained largely indistinguishable regarding the prevalence of cytopenia, LGL counts, co-existing autoimmune diseases, and indications for treatment commencement, as well as immune phenotypes (*p* > 0.05). All patients with a *STAT3* mutation had a CD7+ CD8− CD56+/dim phenotype. None had arthritis or other concurrent autoimmune diseases, while only one had cytopenia (severe anemia).

### 2.4. Treatment and Outcome

**Figure 3:**
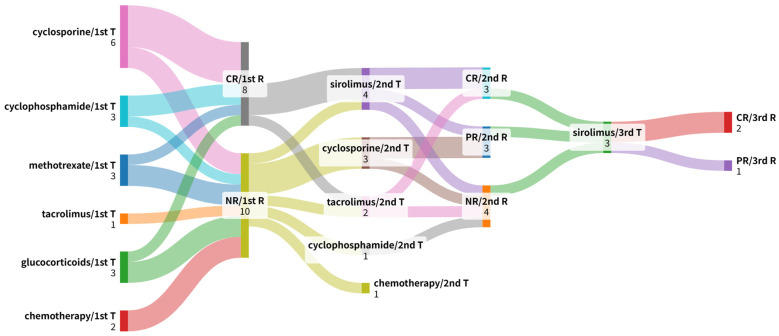
The dynamic longitudinal treatment trajectories and clinical outcomes of NK-LGLL patients, visualized via a Sankey diagram illustrating the transition patterns from first-line treatment (1st T) to subsequent therapies (2nd T, 3rd T) and responses (1st R, 2nd R, 3rd R).

Among the 35 patients, 18 (51.4%) received treatment. As shown in Figure 3 and Table 2, first-line regimens included cyclosporine (CsA *n* = 6), cyclophosphamide (CTX *n* = 3), methotrexate (MTX *n* = 3), tacrolimus (FK506 *n* = 1), glucocorticoids (*n* = 3), and chemotherapy during the time that they were misdiagnosed with PTCL (*n* = 2). Regarding treatment efficacy (Table 2), CsA and CTX demonstrated superior efficacy compared to other agents. In the CsA subgroup, four patients achieved a BOR of CR. Notably, all four CR responders maintained their response until the time of treatment discontinuation or last follow-up (median DOR not reached; range 3.0+ to 46.2+ months). The median TTNT for the CsA group was 11.7 months, with three patients switching to next-line therapy due to adverse events rather than disease progression. In the CTX subgroup (*n* = 3), two patients (66.7%) achieved CR with sustained remission. The DOR for these patients reached 34.4+ and 35.4+ months, respectively, both remaining on treatment at the data cut-off. Only one patient achieved a durable response (DOR 39.1+ months) with MTX, while the remaining two showed no clinical response. The patient who received FK506 did not respond to the treatment. One patient on glucocorticoids achieved CR, and remission was sustained for 6 months. Chemotherapy yielded no responses.

Sirolimus (*n* = 7), CsA (*n* = 3), and FK506 (*n* = 2) were the most commonly used salvage therapies. Sirolimus provided an overall response rate of 85.7% (ORR 85.7%, 95% CI 42.1–99.6%), outperforming FK506 (50.0%) and CsA (66.7%). The median DOR was not reached in sirolimus responders (range 1.0 to 33.4+ months), with a median TTNT of 28.5 months. One responder discontinued treatment due to adverse events.

In patients with concurrent autoimmune diseases, hematological remission was accompanied by improvements in autoimmune-related symptoms. Notably, among the three patients with peripheral neuropathy, CsA, MTX, or CTX was administered as a first-line therapy. All attained hematological remission and experienced relief from neuropathic pain. However, there remained persistent numbness and neurological deficits.

**Figure 4:**
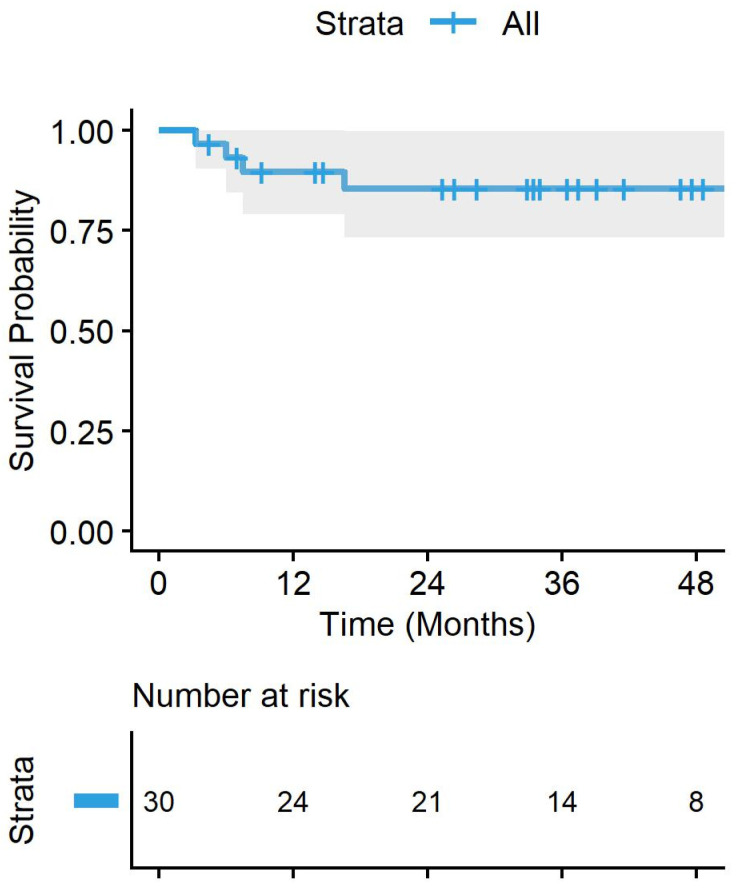
Overall survival of the 35 NK-LGLL patients. The median overall survival (OS) rate in NK-LGLL patients from this cohort was not reached.

After a median follow-up period of 33.4 months (range 3.3 months to 104.9 months), four patients had died—two due to progression of LGLL, one due to severe pneumonia, and one due to co-existing AML. The median overall survival (OS) was not reached, with an estimated 3-year OS rate of 85.6% (95%CI 73.3%, 99.8%) (Figure 4).

## 3. Discussion

As a rare indolent mature lymphoproliferative clonal disorder entity, the largest cohort of patients with NK-LGLL or chronic natural killer lymphoproliferative disorders was reported by Poullot et al., with 70 patients from France, Italy, and the United States [15]. Herein, we report a series of 35 patients with NK-cell large granular lymphocytic leukemia from a single institution in China, aiming to provide complementary data on the clinical and biological features, as well as management, of NK-LGLL. Compared to the Western NK-LGLL cohort, our cohort had a similar indolent disease duration, and approximately half of the cohort was asymptomatic at diagnosis. The prevalence of clinical symptoms, including fatigue and recurrent infections, and the overall incidence of associated autoimmune diseases were similar. However, our cohort had slightly fewer instances of arthritis (2.9% vs. 21%, *p* = 0.08), as well as fewer associated neoplasms (5.7% vs. 24%, *p* = 0.046).

The results from this study extend and confirm the previously reported analyses of cohorts of NK-LGLL or CLPD-NK patients [15,16,17]. As this is an indolent entity, approximately half of the patients were asymptomatic at diagnosis. Meanwhile, 28.6% of the cohort had autoimmune diseases. Peripheral neuropathy is a notable comorbidity, with an incidence of 8.6%, while the published data indicated rates of 3% (2 in 70) [15] and 12.5% (2 in 16) [17]. Frontline immunosuppressive therapy with CsA or CTX induces good remission (CR rates of 66.7% and 66.7%, respectively).

Our data reveal a unique clinical phenotype of NK-LGLL in Chinese patients as compared to previously reported T-LGL leukemia cohorts (listed in Supplementary Table S2) [5,7,11]. NK-LGLL showed a predilection for thrombocytopenia (31.4% vs. 8–19%), but less frequent and severe neutropenia. The low prevalence of concurrent rheumatoid arthritis (2.8%) was consistent with local data [11] but contrasted sharply with Western studies, highlighting potential ethnicity- or lineage-specific clinical divergence.

The clonal expansion of large granular lymphocytes, attributed to activation-induced cell death resistance from constitutive survival signaling, is a well-recognized basis for the pathogenesis of LGLL. *STAT3* and *STAT5b* somatic mutations have been demonstrated to contribute to the constitutive activation of the JAK/STAT3 signaling pathway, as well as maintaining the expansion and survival of LGLs [18,19]. The mutation rate of *STAT3* was 14.3% in our NK-LGLL cohort, similar to the findings from the Poullot study and the Rajala study (12% and 18%, respectively) [15,20], while it was lower than the reported 40–43% for T-LGLL cohorts [20,21]. Some previous studies have suggested that the *STAT3* mutation status is associated with a higher frequency of cytopenia, B symptoms, and rheumatoid arthritis and higher LGL counts [12,19]. The relatively small number of patients sequenced in our cohort could possibly explain the notable absence of concurrent rheumatoid arthritis or cytopenia in *STAT3*-mutated patients. Both *STAT3* and *STAT5b*, the typical driver mutations in Western cohorts, failed to account for the majority of our cases. Our targeted NGS panel provided comprehensive coverage of the entire *STAT3* and *STAT5B* coding sequence, indicating that this absence was likely a biological finding rather than a technical limitation. This discrepancy may reflect underlying genetic heterogeneity in NK-LGLL across different ethnic or geographical populations. Our preliminary finding underscores the need for large-scale, multi-ethnic genomic studies to better characterize the molecular landscape of NK-LGLL.

Furthermore, our targeted sequencing also identified mutations in *ASXL1*, *SF3B1*, and *TET2* in a subset of our patients. These genes are frequently implicated in myelodysplastic syndromes (MDS) and clonal hematopoiesis of indeterminate potential (CHIP). While their presence in NK-LGLL is intriguing, it remains unclear whether they function as primary oncogenic drivers or represent background clonal hematopoiesis. Given that these mutations have been increasingly recognized in various hematological malignancies, we suggest interpreting these findings with caution; they may reflect a pre-existing clonal landscape that could potentially modulate the immune microenvironment, thereby facilitating the selection of *STAT3*-mutated NK-cell clones. Future studies integrating longitudinal tracking of these clones are essential to ascertain their role in the evolutionary trajectory of NK-LGLL.

This finding suggests that the pathogenesis of NK-LGLL involves multiple dysregulated pathways rather than one single recurrent mutation. Larger-scale, independent cohorts are needed to validate this preliminary conclusion.

Given the lack of prospective trials, there is no formal consensus on NK-LGLL treatment. Cyclophosphamide, methotrexate, and cyclosporine are considered as the main first-line options; our findings confirmed that cyclophosphamide and cyclosporine could induce favorable responses. In line with a previous report of a hematological response induced by the mTOR inhibitor sirolimus [22], our study demonstrated that sirolimus as a salvage therapy achieved an overall response rate of 85.7%. This evidence suggests the therapeutic value of targeting the mTOR pathway in NK-LGLL, which warrants further larger-scale study.

The interpretations of our study should be considered in the context of the following limitations. Firstly, there is no single definitive marker for NK-LGLL, making diagnosis inherently challenging. Novel methods such as KIR rearrangement analysis could better detect NK-LGLL clones. However, this was not systematically applied in this cohort. Secondly, the retrospective nature and relatively small sample size of our single-center study restricted the statistical power for formal comparative analyses between treatment regimens. Consequently, our findings on the efficacy of first-line and salvage therapies remain observational and descriptive; larger, prospective multi-center trials are required to validate these findings and establish a standardized treatment consensus. Finally, although our NGS panel covered common cancer-associated genes, the heterogeneity of our findings suggests that other unidentified molecular drivers or epigenetic mechanisms may exist, warranting broader molecular characterization in future research.

## 4. Materials and Methods

### 4.1. Patient Population

This study included 35 patients diagnosed with natural killer cell large granular lymphocytic leukemia between January 2017 and October 2023 at the Department of Hematology in Peking Union Medical College Hospital. Based on the criteria described by Lamy et al. [23], the diagnosis of natural killer cell LGLL required the presence of persistent LGL clones for more than 6 months, characterized by a typical NK-cell immunophenotype (CD2^+^/sCD3^−^/TCRαβ^−^/CD56^+^) on flow cytometry. For cases with circulating LGL counts less than 0.5 × 10^9^/L, diagnosis was confirmed by LGL bone marrow infiltration, along with typical clinical or hematological features. Furthermore, patients with rapidly progressive diseases or aggressive NK-LGL leukemia were excluded from this study.

The baseline clinical characteristics of these patients were retrospectively analyzed, encompassing a comprehensive assessment of peripheral blood smears, flow-cytometric data, bone marrow biopsy findings, and next-generation sequencing (NGS) outcomes where available. CT or PET-CT was performed in several patients, and notable abnormal results were presented. This study was approved by the ethics committee of Peking Union Medical College Hospital (I-23YJ1004).

### 4.2. Treatment, Responses, and Survival

The indications for treatment included severe neutropenia, defined by an absolute neutrophil count of less than 0.5 × 10^9^/L; moderate neutropenia accompanied by recurrent infections; transfusion-dependent anemia; and/or concurrent autoimmune disorders necessitating therapeutic intervention.

Treatment efficacy was first evaluated at the 3-to-4-month landmark following the commencement of therapy, with the following criteria [9]. A complete hematological response (CR) was characterized by the normalization of blood cell counts along with the disappearance of circulating large granular lymphocytes. A partial response (PR) was indicated by a significant improvement in blood cell counts that did not meet CR criteria—specifically, hemoglobin levels exceeding 80 g/L, platelet counts surpassing 50 × 10^9^/L, and neutrophil counts above 0.5 × 10^9^/L, without the need for transfusion or growth factors. Non-response (NR) was defined as the failure to achieve either CR or PR, or transfusion dependence. Treatment failure was identified by NR or the presence of disease progression (over 50% increase in absolute LGL count, decrease in blood cell counts, or new-onset symptomatic organomegaly).

The overall survival analysis included all patients, with the survival time defined as the interval from the date of diagnosis to the date of the last follow-up or death due to any cause. Among patients receiving treatment, the best overall response (BOR) was determined as the highest response level achieved at any time point during the follow-up period, categorized as CR, PR, or NR. TTNT was defined as the interval between the initiation of the current therapy and the date of the subsequent treatment line or death from any cause. The duration of response (DOR) was measured from the date of the first documented objective response (CR or PR) until the date of disease progression or death from any cause.

### 4.3. Flow Cytometry and Next Generation Sequencing (NGS)

Flow cytometry was performed on the FACS Canto II flow cytometer (BD) with the following markers for diagnosis and differential diagnosis: CD2, CD3, CD4, CD5, CD7, CD8, CD10, CD11c, CD16, CD19, CD20, CD22, CD23, CD25, CD34, CD38, CD56, CD57, CD103, CD123, CD138, CD148, Cyclin D1, FMC7, Kappa, Lamda, IgG1, surface IgM, TCRαβ, and TCRγδ.

The sequencing experiment included the exonic regions of genes associated with myeloid tumors, lymphoid tumors, histiocytic disorders, and common tumor pathways (JAK-STAT, MAPK, PI3K-ALK-mTOR). A detailed list of genes sequenced is provided in Supplementary Table S1. DNA samples were extracted from peripheral blood or bone marrow samples; then, DNA libraries were constructed using adapters with UMI error correction tags and enriched using hybridization probes. Targeted gene sequencing of the DNA libraries from the samples was performed on the MGISEQ-2000 (MGI, Shenzhen, China) sequencing platform. This allowed the analysis of SNV and indel variations in the exonic regions of the genes included in the panel, with sequencing data ranging from 3 to 4 GB. Sequencing data were aligned to the GRCh37/hg19 reference genome using the BWA software (version 0.7.11). Variant analysis was conducted with TNscope (Sentieon ^®^ Genomics, CA, USA). The mean sequencing depth of the targeted regions was 2000×. To ensure high confidence in somatic variant calling, we applied a stringent filtering strategy. Variants were retained only if they met the following criteria: (1) the site-specific read depth was at least 10% of the panel’s mean sequencing depth; (2) the variant was supported by a minimum of 9 mutant reads.

### 4.4. Statistical Analysis

Statistical analyses were performed using the SPSS software (version 22.0, IBM). Descriptive statistics were expressed as the median and range for continuous data, while categorical data were presented as numbers and percentages. *p*-values were calculated using Fisher’s exact test for categorical variables and the Mann–Whitney U test for continuous variables. Overall survival (OS) was estimated using Kaplan–Meier analysis. A significance level of *p* ≤ 0.05 was deemed statistically significant.

## 5. Conclusions

In this study, we describe our real-world experience (clinical features, immunophenotypes, mutation status, treatment choices, and response to therapy) regarding 35 unselected patients with NK-cell large granular lymphocyte leukemia who were diagnosed and followed up with at a single institution in China. As the largest retrospective Asian NK-LGLL cohort, they presented similar clinical manifestations to Western patients, with fewer co-existing malignancies and fewer cases of arthritis. Cyclosporine and cyclophosphamide provided substantial responses at the frontline, while sirolimus showed potential as a salvage therapy. These findings contribute to a better understanding of this rare indolent NK-cell proliferative disorder. Future multi-center, prospective trials are needed to evaluate the role of sirolimus in NK-LGLL treatment.

## Figures and Tables

**Table 1:** Patient baseline characteristics.

Variable	Number (%) (*n* = 35)
Age, median (range), years	58 (30–78)
Diagnosis before age 50	8 (22.9%)
Gender	
Female	16 (45.7%)
Male	19 (54.3%)
LGL count at diagnosis (10^9^/L), median (range)	3.9 (0.11–114.8)
Median absolute neutrophil count, 10^9^/L (range)	1.9 (0.12–10.7)
Median lymphocyte percentage, (range)	65% (12–99%)
Median hemoglobin, g/dL (range)	12.9 (4.5–16.3)
Median platelet count, 10^9^/L (range)	198 (6–453)
Hematologic manifestations (%)	
Neutropenia (<1.5 × 10^9^/L) (%)	11 (31.4%)
Severe neutropenia (<0.5 × 10^9^/L) (%)	3 (8.6%)
Anemia (<11 g/dL) (%)	15 (42.9%)
RBC transfusions at presentation (%)	6 (17.1%)
Thrombocytopenia (<100 × 10^9^/L) (%)	11 (31.4%)
Platelet < 50 × 10^9^/L (%)	6 (17.1%)
Splenomegaly (%)	3 (8.6%)
Hepatomegaly (%)	1 (2.9%)
Fatigue (%)	7 (20.0%)
Bleeding (%)	2 (5.7%)
Autoimmune conditions (%)	10 (28.6%)
Autoimmune hemolytic anemia	2 (5.7%)
Systemic lupus erythematosus	2 (5.7%)
Primary biliary cholangitis	1 (2.9%)
Vasculitis	1 (2.9%)
Rheumatoid arthritis	1 (2.9%)
Immune-mediated peripheral neuropathy	3 (8.6%)
Recurrent infections (%)	7 (20.0%)
Pneumonia	6 (17.1%)
Virus infection	1 (2.9%)
Co-existing malignancies (%)	2 (5.7%)
prostate cancer	1 (2.9%)
MDS-EB	1 (2.9%)
Percentage of patients treated (%)	18 (51.4%)

**Table 2:** Treatment responses in NK-LGLL patients.

	First-Line Therapy Best Overall Response		Salvage Therapy Best Overall Response	
Cyclosporine	CR	4/6, 66.7%	CR	0
	PR	0	PR	2/3, 66.7%
	NR	2/6, 33.3%	NR	1, 33.3%
	ORR	66.7% (95%CI 22.3–95.7%)	ORR	66.7% (95%CI 9.4–99.2%)
Cyclophosphamide	CR	2/3, 66.7%	CR	0/1, 0
	PR	0	PR	0/1, 0
	NR	1, 33.3%	NR	1, 100%
	ORR	66.7% (95%CI 9.4–99.2%)	ORR	0 (95%CI not reliable)
Methotrexate	CR	1/3, 33.3%	NA	
	PR	0		
	NR	2/3, 66.7%		
	ORR	33.3% (95%CI 0.8–90.6%)		
Tacrolimus	CR	0/1, 0	CR	1/2, 50.0%
	PR	0/1, 0	PR	0/2, 0
	NR	1, 100%	NR	1/2, 50.0%
	ORR	0 (95%CI not reliable)	ORR	50% (95%CI not reliable)
Sirolimus	NA		CR	4/7, 57.1%
			PR	2/7, 28.6%
			NR	1/7, 14.3%
			ORR	85.7% (95%CI 42.1–99.6%)

ORR: overall response rate (CR + PR); 95% CI: Clopper–Pearson confidence interval. CI was not calculated for groups with *n* < 3 or insufficient outcome data due to the high degree of statistical uncertainty. NA not applicable.

## Data Availability

The data that support the findings of this study are available from the corresponding author Y.Z. upon reasonable request.

## Supplementary Materials

The following supporting information can be downloaded at https://www.mdpi.com/article/10.3390/ijms27146227/s1.
