# Supplementary material for: Clinical Manifestations and Genetic Profile of Chinese Patients with NK-Cell Large Granular Lymphocytic Leukemia—A Single-Center Retrospective Analysis
Source: Int J Mol Sci. 2026 Jul 13;27(14):6227. doi: 10.3390/ijms27146227 (PMC13410105; doi:10.3390/ijms27146227)
Supplement: Supplementary file 1 [file ijms-27-06227-s001.zip › ijms-4329707-supplementary.pdf]

Supplementary Table S1

**List of genes sequenced.**
ABL1,ACD,ACTB,AKT1S1,AKT2,ALK,ARAF,ARID1A,ARID1B,ARID5B,ASXL1,ASXL2,ASXL3,ATG2B,ATM,ATR,B2M,BCL11B,BCL2,BCOR,BCORL1,BIRC3,BIRC6,BLM,BRAF,BRCA1,BRCA2,BRIP1,BTG1,BTG2,BTK,CAD,CALR,CARD11,CASP10,CASP8,CBL,CCND1,CCND2,CCND3,CD36,CD58,CD70,CD79B,CDK12,CDK4,CDKN1B,CDKN2A,CEBPA,CHD2,CHD3,CHD8,CHEK2,CIC,CP,CREBBP,CSF1R,CSF3R,CTC1,CXCR4,DCAF6,DDX10,DDX3X,DDX41,DKC1,DNAJC21,DNMT3A,DTHD1,DUSP2,DUSP4,EFL1,EGFR,EGR2,ELANE,EOMES,EP300,EPCAM,EPHA5,ERBB2,ERBB3,ERBB4,ETNK1,ETS1,ETV6,EZH2,FAM5C,FANCA,FANCC,FAS,FASLG,FBXW7,FGFR2,FGFR3,FGFR4,FLT3,FLT4,FOXO1,G6PC3,GADD45B,GATA1,GATA2,GFI1,GNA13,GNAI2,GNAS,GSKIP,HAX1,HIST1H1E,HRAS,ID3,IDH1,IDH2,IKBKB,INO80,INPP4B,INPP5D,INTS8,IRF4,IRF8,IRS2,ITPKB,JAK2,JAK3,KDM5A,KDM6A,KDR,KIT,KLHL14,KLHL6,KMT2A,KMT2D,KRAS,LIPE,LRP1B,LZTR1,MAF,MALT1,MAP2K1,MAP2K2,MAP3K1,MAPK1,MAPK14,MAPK3,MAPK8,MBD4,MCL1,MECOM,MED12,MEF2B,MET,MGA,MGAM,MLH1,MLL2,MLL3,MLL4,MLST8,MPL,MSH2,MSH6,MTOR,MYC,MYD88,NAF1,NF1,NFKB2,NHP2,NOP10,NOTCH1,NOTCH2,NPM1,NRAS,NTRK1,PARN,PAX5,PCLO,PDGFA,PDGFRA,PDGFRB,PDPK1,PHF6,PHLPP1,PIK3CA,PIK3CD,PIK3R2,PIM1,PKN3,PLCG1,PMS2,POT1,PPM1D,PPP2R2D,PPP6C,PRDM1,PRKCQ,PTEN,PTPN1,PTPN11,PTPN6,PTPRD,RAF1,RB1,RELN,RET,RHEB,RHOA,RICTOR,RIPK1,ROS1,RPTOR,RTEL1,RUNX1,SAMD9,SAMD9L,SAMHD1,SBDS,SCN1A,SETBP1,SETD1B,SETD2,SF3B1,SGK1,SH2B3,SIN3A,SMARCA4,SMARCB1,SMC1A,SMC3,SOCS1,SOX10,SOX11,SPEN,SRP72,SRSF2,STAG2,STAT3,STAT5B,STAT6,STK11,TBL1XR1,TCF3,TERC,TERT,TET1,TET2,TGM7,TINF2,TLR8,TNFAIP3,TNFRSF14,TP53,TP73,TRRAP,TSC1,TSC2,U2AF1,UBA1,UNC13D,UNC5B,VCL,VEGFA,WDR24,WHSC1,WRAP53,WT1,XPC,XPO1,YWHAE,ZCCHC8,ZEB1,ZMYM3,ZNF292,ZNF608,ZRSR2

Supplementary Table S2
Comparison of our NK-LGLL cohort and Western NK-LGLL cohort.

	Our study (N=35)	Poullot et al (N=70)	p value
Age, median (range), years	58 (30-78)	61 (23–82)	0.31
Sex ratio M/F(%men)	19/16 (54.3%)	41/29 (58.6%)	0.84
Symptoms at diagnosis (%)	18 (51.4%)	36 (51.4%)	1.00
Fatigue (%)	7 (20.0%)	18 (25.7%)	0.64
Tumoral syndrome	4 (11.4%)	18 (25.7%)	0.13
Splenomegaly (%)	3 (8.6%)	10 (14.3%)	0.74
Hepatomegaly (%)	1 (2.9%)	9 (12.9%)	0.23
Polyadenopathy	0	4 (5.7%)	0.57
Autoimmune conditions (%)	10 (28.6%)	27 (38.6%)	0.35
Autoimmune cytopenia	2 (5.7%)	10 (14.3%)	0.32
Systemic lupus erythematosus	2 (5.7%)	0	0.11
Primary biliary cholangitis	1 (2.9%)	0	0.33
Vasculitis	1 (2.9%)	3 (4.3%)	1.00
Arthritis	1 (2.9%)	11 (15.7%)	**0.08**
Peripheral neuropathy	3 (8.6%)	2 (2.9%)	0.29
Recurrent Infections (%)	7 (20.0%)	10 (14.3%)	0.35
Pneumonia	6 (17.1%)	-	-
Virus infection	1 (2.9%)	-	-
Co-existing malignancies	2 (5.7%)	17 (24.3%)	**0.046**

All P-values are nominal and have not been adjusted for multiple testing.

Supplementary Table S3 Somatic mutations in NK-LGLL patients

Study ID	Gene	Transcript	Exon	Genomic Variant	Amino Acid Change	VAF (%)
1	STAT3	NM_003150	exon21	c.1940A>T	p.Asn647Ile	24.9%
12	no mutation detected					
28	CD36	NM_000072	exon4	c.268C>T	p.Pro90Ser	44.3%
28	TET2	NM_001127208	exon11	c.4824T>A	p.Tyr1608*	6.4%
29	STAT3	NM_003150.4	exon20	c.1842C>G	p.Ser614Arg	1.8%
30	no mutation detected					
31	no mutation detected					
32	NOTCH2	NM_024408.4	exon18	c.2816C>T	p.Pro939Leu	48.4%
35	TP53	NM_000546.6	exon7	c.713G>A	p.Cys238Tyr	77.0%
35	BCORL1	NM_001184772.3	exon15	c.5212C>T	p.Gln1738*	71.2%
35	BCOR	NM_001123383.1	exon4	c.1446dupT	p.Pro483Serfs*16	63.5%
35	SRSF2	NM_003016.4	exon1	c.284C>G	p.Pro95Arg	34.0%
35	ASXL1	NM_015338.6	exon12	c.1927dupG	p.Gly646Trpfs*12	33.5%
35	PTPN11	NM_002834.5	exon3	c.214G>A	p.Ala72Thr	11.8%
5	no mutation detected					
20	XPO1	NM_003400	exon15	c.1712A>T	p.Glu571Val	1.10%
27	EP300	NM_001429	exon6	c.1519A>G	p.Ser507Gly	1.50%
27	TNFAIP3	NM_001270507	exon3	c.380T>G	p.Phe127Cys	1.30%
27	MYD88	NM_002468	exon4	c.695T>C	p.Met232Thr	1.60%
2	no mutation detected					
6	no mutation detected					
7	no mutation detected					

11	SF3B1	NM_012433	exon15	c.2099A>G	p.Lys700Arg	22%
15	GNA13	NM_006572	exon4	c.736_737insA	p.Leu246Hisfs*6	11.50%
26	no mutation detected					
4	no mutation detected					
18	STAT3	NM_003150	exon21	c.1981G>T	p.Asp661Tyr	16.4%
18	ASXL1	NM_015338	exon12	c.1927dupG	p.Gly646Trpfs*12	2.1%
21	no mutation detected					
22	no mutation detected					
